# B-Cell-Based Immunotherapy: A Promising New Alternative

**DOI:** 10.3390/vaccines10060879

**Published:** 2022-05-31

**Authors:** Sneh Lata Gupta, Naeem Khan, Srijani Basu, Vijay Soni

**Affiliations:** 1 National Institute of Immunology, New Delhi 110067, India; snehlata34400@gmail.com; 2 Center for Immunobiology and Department of Investigative Medicine, Western Michigan University Homer Stryker M.D. School of Medicine, Kalamazoo, MI 49007, USA; 3 Department of Medicine, Weill Cornell Medicine, New York, NY 10065, USA; 4 Division of Infectious Diseases, Weill Department of Medicine, Weill Cornell Medicine, New York, NY 10065, USA

**Keywords:** B cells, IgM, IgG, B cell receptor, Breg

## Abstract

The field of immunotherapy has undergone radical conceptual changes over the last decade. There are various examples of immunotherapy, including the use of monoclonal antibodies, cancer vaccines, tumor-infecting viruses, cytokines, adjuvants, and autologous T cells carrying chimeric antigen receptors (CARs) that can bind cancer-specific antigens known as adoptive immunotherapy. While a lot has been achieved in the field of T-cell immunotherapy, only a fraction of patients (20%) see lasting benefits from this mode of treatment, which is why there is a critical need to turn our attention to other immune cells. B cells have been shown to play both anti- and pro-tumorigenic roles in tumor tissue. In this review, we shed light on the dual nature of B cells in the tumor microenvironment. Furthermore, we discussed the different factors affecting the biology and function of B cells in tumors. In the third section, we described B-cell-based immunotherapies and their clinical applications and challenges. These current studies provide a springboard for carrying out future mechanistic studies to help us unleash the full potential of B cells in immunotherapy.

## 1. Introduction

In recent years, immunotherapy has made extraordinary advances and brought longstanding survival benefits to patients with cancer. However, many patients do not respond to immunotherapy, or their responses are temporary, indicating immune resistance. While a lot has been achieved in the field of T-cell immunotherapy, much less has been spoken about the role of its contemporary—B cells in the tumor microenvironment. Most immunotherapies at present target T cells through checkpoint inhibitors and mechanistically work by reactivating anti-tumor immunity [1]. T cells can mediate their tumor-killing function directly, and immunotherapy strategies that take advantage of this include CAR-T-cell therapy, checkpoint inhibitors, and T-cell-based cancer vaccines. T cells can also mediate tumor killing indirectly via cytokines (cytokine therapy), monoclonal antibodies, oncolytic viruses, and adjuvants [2,3].

Of late, immunotherapy based on immune checkpoint blockade has garnered a lot of attention [1]. However, these therapies have limitations, such as a low frequency of mutation, reduced immune cell infiltration in tumors, and the suppressive nature of the tumor microenvironment [4], and, therefore, there is a need for an alternative approach using other immune cells.

**Figure 1:**
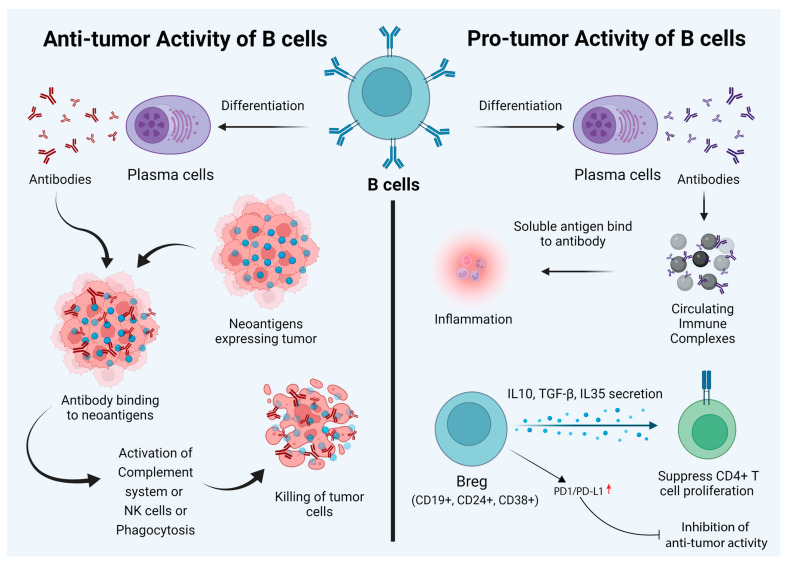
Dual nature of B cells in the cancer tumor microenvironment. Their anti-tumor characteristics can be utilized to empower immunotherapy goals. While behaving as anti-tumorigenic (left panel), B cells can recognize tumor-specific “neoantigens” and can stimulate antibody production, thus killing oncogenic cells. B cells can also have a pro-tumorigenic effect and promote tumor growth. Circulating immune complexes (CICs) and specific types of B cells (e.g., CD19+, CD24+, and CD38+) are the main factors behind this. These Breg cells differentiate due to inflammation and various other factors. They are responsible for immune tolerance and enhance Foxp3 expression in Treg cells. However, in some hepatocellular carcinomas, the expression of PD1/PD-L1 can suppress the anti-tumor activity of Bregs.

Bursa-derived lymphocytes (B cells) can generate immunoglobulins (antibodies) and play key roles in humoral immunity. Generally, B-cell receptors (BCRs) identify antigens, and this leads to B-cell activation and differentiation into plasma cells (Figure 1) [5]. B cells can be divided into three categories: (i) B1B cells, present in the pleural cavities and peritoneum; (ii) follicular B (FOB) cells or B2 B cells, found in the lymph nodes, spleen, and Peyer’s patches; and (iii) marginal zone B (MZB) cells, located in the marginal sinus of the spleen [5]. Both B1B cells and FOB cells produce antibodies with a high affinity and specificity. MZB cells produce antibodies for blood-borne pathogens in an early phase of infection, their mostly T-independent antibody response, and a low-affinity IgM antibody [6,7]. Naïve B cells activate upon interactions with their cognate receptors, generate extrafollicular responses in the early course of infection, and differentiate into short-lived plasma cells. Later on, a few B cells undergo the germinal center reaction and differentiate into either long-lived plasma cells or memory B cells. Plasma cells secrete antibodies [8]. More importantly, B cells also secrete cytokines that can affect T-cell function, dendritic cell (DC) function, and lymphoid tissue reorganization [9,10]. 

The use of immunotherapeutic interventions based on B cells and T cells would be an effective method of combating tumors. Furthermore, there is evidence that B cells infiltrate into tumor tissues; such B cells are called tumor-infiltrating B (TIB) cells, and these cells can differentiate into other B-cell subtypes [11]. Regulatory B cells (Bregs) are part of TIBs and have a direct association with tumor immunosuppression [2]. TIBs can modulate the immune response through interactions with other immune cells, such as Treg cells, NK cells, and CD4+ T cells [12]. Reports suggest that B cells play an important role in various cancers, such as breast cancer [13,14,15], epithelial ovarian cancer [16], melanoma [17], non-small-cell lung cancer [18,19], and renal cell carcinoma [20]. 

In this review, we summarized recent advances in the potential role of B cells in tumor immunity and immunotherapy. We begin with discussing the dual role of B cells in the tumor microenvironment, followed by a review of the different factors that affect B-cell function in tumor immunity, and lastly, we conclude with the various immunotherapies based on B cells, their clinical application, and challenges. 

## 2. Dual Role of B Cells

The role of B cells in tumor immunotherapy is controversial and is not widely discussed compared to that of T cells. Based on existing reports, it is safe to say that B cells play a dual role in cancer immunotherapy. Besides secreting antibodies, B cells also regulate T cells and innate immune cell responses. B cells process and present antigens, and the balance of B-cell subtypes and their functions affect pro- and anti-tumorigenic functions [21]. Given this background, it is important to note that there are disparate reports regarding the prognostic value of B cells in tumor immunity. Here, we provide a summary of the role of B cells in pro- and anti-tumor immunity (Figure 1). 

## 3. Anti-Tumor Activity of B Cells

The antibody response in the tumor microenvironment is triggered by the expression of specific new antigens called neoantigens (as a result of mutations); the overexpression of genes; aberrant post-translational modifications; the expression of a specific differentiation marker, for example, CD20 in leukemia; and the expression of a marker normally found in other tumors, for example, the expression of cancer–testis antigens in melanoma and other tumor types [22]. An important thing to note is that CD20 is not the exclusive marker for leukemic cells but is also expressed in normal B cells from the stage of pro-B cells. CD20 also functions as a B-cell co-receptor to modulate the levels of B-cell signaling via Src-family kinases [23].

**Figure 2:**
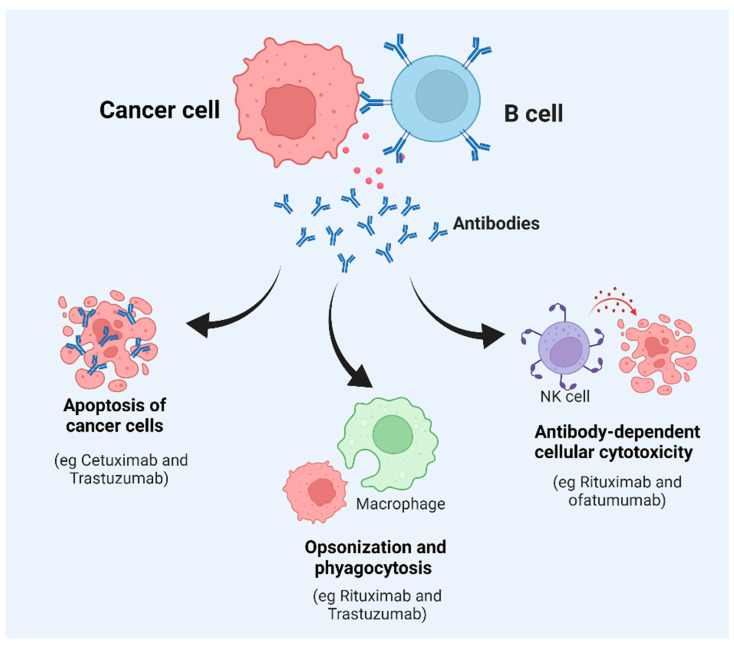
Key factors affecting B-cell function and tumor survival. Once a B cell interacts with tumor cells, it can generate different types of responses, including the secretion of antibodies. This can result in apoptosis, phagocytosis, opsonization, or direct killing of the target cancerous cells or tumors.

Antibodies produced by B cells can lead to the killing of tumor cells through the activation of the complement system, direct killing by NK cells, or phagocytosis by macrophages [24] (Figure 2). There are several studies to suggest that the antibodies produced by B cells against tumor cells lead to the efficient control of tumor growth. For example, a study conducted by Li et al. showed that an injection of tumor-specific antibodies leads to complement activation and tumor regression in a model of large-cell lung carcinoma [25]. Carmi et al., using an allogenic tumor model, showed that antibodies produced by B cells activate dendritic cells, which, in turn, activate the cytotoxic T-cell response leading to the control of tumor growth [26,27]. B cells can also directly target tumor cells. Tao et al. showed that CD19+ B cells from tumor-draining lymph nodes express FAS ligand (FAS-L) and, upon interaction with FAS, lead to the apoptosis of 4T1 murine breast cancer cells [28]. Another example of direct killing mediated by B cells is that of CpG-activated B cells, which can directly target cancer cells through the TRAIL/Apo-2L pathway [29] (Figure 2). B cells can also produce granzymes upon IL-21 stimulation and can mediate the killing of non-stimulated ones [30]. In addition, B cells that express B220, CD19, and CD11c can act as antigen-presenting cells (APCs). In ovarian cancers, B cells have been found in close proximity to T cells, indicating that they can act as APCs [31]. The role of B cells has also been studied in non-small-cell lung cancer, where B cells have been found to act as APCs to CD4+ T cells. Activated B cells classified as CD69^+^ HLA-DR^+^ CD27^+^ CD21^+^ could induce Th-1 differentiation, whereas exhausted B cells categorized as CD69^+^ HLA DR^+^ CD27^−^ CD21^−^ induced the generation of Tregs [32].

## 4. The Pro-Tumor Activity of B Cells

While B cells can mediate tumor cell killing, these cells can also promote tumor growth. Circulating immune complexes (CICs) are made of antibodies attached to multiple soluble antigens. CICs are formed by IgG antibodies bound to tumor antigens, such as globulin, viral RNA antigens released from cell debris, and apoptotic and necrotic cells. 

These complexes can induce inflammation by recognizing the Fc region [33]. In a model of epithelium carcinogenesis (K14-HPV16 mice), it was shown that CICs produced by B cells induced chronic inflammation through the activation of myeloid cells via engagement of the FcR (Figure 1). Antibody production in the tumor-draining lymph nodes of melanoma-bearing mice augments tumor growth [34]. Tumor-promoting abilities are also maintained by diverse B-cell populations known as regulatory B cells or Bregs. Breg cells are a subpopulation of B-cells (both mature and immature) with immunoinhibitory abilities. There are multiple factors playing a role in the development of Breg cells, such as CD40, activation, inflammation, Toll-like receptors (TLRs), and various transcription factors (Figure 1) [35]. These cells mediate immune tolerance and are defined as CD5^+^ CD24^hi^ CD27^+^ CD38^hi^ B cells [36]. Bregs produce IL-10, IL-35, and TGF-β. Bregs suppress CD4+ T-cell proliferation and lead to Foxp3 expression in Tregs by producing IL-10, IL-35, and TGF-β. In patients with acute myeloid leukemia (AML), Bregs are categorized as CD19^+^, CD24^+^, and CD38^+^, and the presence of these cells is correlated with poor prognosis [37] (Figure 1). Moreover, it has been reported that the expression of checkpoint inhibitors, such as PD-1 and PD-L1, by Bregs in hepatocellular carcinoma (HCC) samples leads to the suppression of anti-tumor activity [38] (Figure 1).

There are several studies that have used mice models to study cancer. Fibrosarcoma cells when injected into B6 mice showed pro-tumor activity, while the depletion of B cells via the administration of anti-IgM antibodies in a xenograft mice model led to a reduction in tumor growth, metastasis, and anti-tumor activity [12]. The administration of human papillomavirus type 16 (HPV-16) transgenic cells into Rag-/- mice and CD4-/- or CD8-/- mice led to a reduction in skin tumor growth [39]. The transfer of B16/F10 cells into B6 mice enhanced melanoma [12]. Cancers such as EL4 thymoma, MC38 colon carcinoma in B6 mice, and EMT-6 breast carcinoma in BALB/c mice led to a reduction in tumor growth in mice lacking B cells compared to wild-type mice [40]. Mouse models have been used to study various cancer types. Conditional mouse models harboring floxed-Myd88^L252P^ CD19-Cre and Myd88^L252P^-IRES-Yfp; CD19-Cre have been used to study plasma cell neoplasms. To understand the role of TRAF3 in cancer, TRAF3-deficient mice and TRAF3xBCL2 tg mice have been used, as they develop a distinct type of cancer. Similarly, c-myc-driven mouse models have been used to study lymphoma. *Eµ-TCL-1* transgenic mice and *Traf2*DN/*BCL2*-double-tg mice have been used in a chronic lymphocytic leukemia (CLL) study [41]. 

The above examples shed light on the duality of B cells in cancers. Antibodies can be pro-tumorigenic when they form circulating immune complexes (CICs). CICs bind to Fcγ receptors on immunosuppressive myeloid cells and promote angiogenesis. However, antibodies have also shown anti-tumorigenic function. Antibodies against tumor antigens show Fc-mediated effector functions, such as complement-dependent cytotoxicity (CDC), ADCC, FcR-driven phagocytosis, and antigen presentation by dendritic cells [27].

Similarly, FAS/FAS-L interaction also shows both pro- and anti-tumor activities. The FAS/FAS-L interaction in Bregs induces apoptosis in CD4^+^ T cells, while the same interaction also helps in killing tumor cells. Granzyme B also shows dual activity. It causes pro-tumorigenic activity by degrading the T-cell receptor (TCR) ε chain without apoptosis; however, it induces apoptosis in B-CLL cancer [42]. 

**Mechanistic insights into the role of B cells in Immunotherapy**

The role of B cells in immunotherapy is rather controversial and complicated. Depending on the state of activation, B cells have been reported to play divergent roles in T-cell differentiation and effector functions in various tumor models. B cells can execute their regulatory functions through the release of cytokines. One of the mechanisms by which B cells exert their effect is through the production of IL-10 and their interaction with Tregs. However, all of these studies have been performed in mice that lack B cells. Using wild-type mice, DiLillo et al. demonstrated that B cells are essential for optimal CD4^+^ and CD8^+^ immunity induction. Specifically, in a B16 melanoma model, tumor growth increased in a B-cell-depleted host. 

As opposed to resting B cells, several reports have denoted the importance of activated B cells in cellular immunotherapy. Many of these reports focus on the role of activated B cells as effective antigen-presenting cells (APCs) for T cells. It was recently reported that the adoptive transfer of activated B cells specific for 4T1 tumors into tumor-bearing hosts resulted in the initiation of T-cell-mediated immunity to 4T1 tumors in the peripheral blood and the spleen. Furthermore, B cells play a major role in the production of antibodies specific to tumor-associated epitopes. Antibodies exert their anti-tumor effects by various means. One mechanism is antibody-dependent cell-mediated cytotoxicity (ADCC), which is mediated by neutrophils, T cells, macrophages, and natural killer cells. Another mechanism for tumor lysis involves complement-dependent cytotoxicity (CDC). While antibodies by themselves are not effective in causing lysis of target cells, antibodies of the IgM and IgG classes can activate the complement system and cause cell lysis [43]. 

However, B-cell-mediated tumor immunity is further complicated by tumor evasion strategies. Tumors under ADCC attack develop mechanisms to evade NK cell attack. A well-known evasion strategy is through the shedding of the endogenous MHC-class-I-related chain molecule (MIC), which binds the activation receptor NKG2D on NK cells and results in the internalization of NK2GD and reduced NK cell activity. Therefore, the shedding of MIC has been established as a mechanism to evade NK cell immunosurveillance.

A recent study found that, in tumors with *MICA* amplification, the presence of high levels of IgG1/3 B cells was associated with better survival in breast cancer and melanoma. In sharp contrast, the levels of IgG1/3 level did not influence survival for tumors without *MICA* amplification. These results suggest intricate interactions between B-cell-mediated immune responses and tumor ADCC pathway defects [44].

**Factors affecting B-cell function in the tumor microenvironment**

As described previously, B cells can have both tumor-promoting and anti-tumorigenic effects. Multiple factors in the tumor microenvironment influence B-cell function, such as immune cells, the direct action of tumor cells on B cells, immune checkpoint stimulation on B cells, and hypoxia [42].

### 4.1. Immune Cells

B cells present in the microenvironment of a solid tumor express granzyme B and are located close to IL21-secreting Treg cells. IL21 induces granzyme-B-expressing human Breg cells [45]. Moreover, in vitro studies have shown that activated CD4^+^ CD25^+^ Tregs can suppress B-cell proliferation by inducing granzyme-dependent cell death [46]. MDSC can induce Bregs in a mouse model of breast cancer. These Bregs express PD-L1 and PD-1. MDSCs can impair B-cell function through the secretion of IL-7, which is correlated with reduced antibody production [47].

### 4.2. Cytokines and Metabolites

Metabolites such as leukotriene B4 (LTB4) can activate the peroxisome proliferator-activated receptor α (PPARα) in B cells inducing Breg differentiation [48]. Human breast cancer cells also express the CXCL-13 receptor, which induces the migration of CXCR-5-expressing B cells, and the coculture of CXCR-5-expressing B cells with cancer cells such as MCF-7 can induce the apoptosis of B cells and the appearance of a Breg population [49].

### 4.3. Expression of Immune Checkpoint on B Cells

Checkpoint inhibitors (CPIs) work via T-cell modulation. Various mAbs have been approved till date, including Ipilimumab (anti-CLTA-4), Pembrolizumab (anti-PD-1), Nivolumab (anti-PD-1), and Atezolizumab (anti-PD-L1) that function through inhibiting the interaction of the checkpoint inhibitor with their ligands [50]. PD-1 expression on B cells prevents signal transduction through the B-cell receptor (BCR) via the recruitment and phosphorylation of protein tyrosine phosphatase non-receptor type 11 (PTPN11). PTPN11 deactivates spleen tyrosine kinase (SYK), preventing the downstream signaling cascade [51]. 

### 4.4. Hypoxia

Hypoxia is a hallmark of cancer. Due to intense cell proliferation, as well as immune cell infiltration, there is vascular disorganization leading to hypoxia and the activation of hypoxia-inducible factors (HIFs) at the tumor site. Reports indicate that the deletion of glucose transporter 1 (Glut1), a target of HIF-1α, leads to reduced B-cell proliferation and decreased antibody production capacities [52].

## 5. B-Cell-Based Immunotherapy and Their Clinical Applications

With new developments in tumor immunity over the last decade, it is clear that B cells play an important role in tumor biology. Despite the challenges, researchers have redefined the role of B cells in cancers and made them prominent, next-generation candidates for tumor immunotherapy. Tumor antigens can stimulate B cells to produce tumor-specific IgG-dependent antibodies [53,54], thus imprinting our body with a long-lasting immune memory against it. B cells are also found to stimulate other components of the tumor-immune system, such as promoting Th1 cells, activating cytotoxic T-cells, and secreting cytokines [24,42]. Therefore, there are various categories of B-cell-based immunotherapies.

The currently available B-cell-based immunotherapies and the clinical trials that have been completed are shown in Table 1 as per the clinical.gov website.

Bregs produce tumorigenic cytokines, such as IL-35, TGF-β, and IL-10, which makes the microenvironment conducive for tumor progression. The treatment of nine CLL patients with autologous B cells expressing human CD40 and IL-2 led to the activation of T cells and increased IFN-γ, granzyme B, and IL-5 production [55]. A detailed description of the different types of B-cell-based immunotherapies, their applications, and limitations are discussed below.

### 5.1. Monoclonal Antibody (mAb)

The usage of mAbs in cancer therapy is one of the most used therapies along with conventional therapy, such as radiation, chemotherapy, and surgery. Various mechanisms are involved in the killing of a tumor cell [56]. One of them is antibody-dependent cell cytotoxicity (ADCC), where NK cells release cytotoxic granules, and the other is complement-dependent cytotoxicity (CDC), where a classical complement cascade is involved, for example, Rituximab and Ofatumumab for CLL treatment by CDC (Figure 2). The second mechanism is phagocytosis, which is also called antibody-dependent cell phagocytosis (ADCP), where macrophages and/or neutrophils carry out Fc-receptor-mediated endocytosis of tumor cells (Figure 2). Rituximab and Trastuzumab also mediate ADCP activity. Another mechanism of tumor killing is apoptosis (Figure 2). This is either through antibody-mediated receptor blocking or ligand blocking. Cetuximab is an anti-EFGR mAb that mediates receptor blocking and its dimerization [57,58]. Trastuzumab is used in the treatment of breast cancer and acts as an anti-HER-2 mAb. It acts on the HER-2 receptor and is an FDA-approved mAb [59,60]. The depletion of B cells with humanized anti-CD20 (Rituximab) treatment is useful in B-cell lymphoma and has limited success in solid tumors. The use of Ibrutinib as a Bruton tyrosine kinase (BTK) inhibitor has shown success in the treatment of pancreatic ductal adenocarcinoma [42]. Obinutuzumab (Gazyva, Genentech) targets CD20 and is approved by the FDA for the treatment of CLL and follicular lymphoma. Ofatumumab (Arzerra, Novartis) leads to B-cell activation and is also approved by the FDA for CLL treatment. Some of the side effects of using these mAbs are toxicity; for example, Alemtuzumab (Campath), a mAb that targets CD52, has been shown to cause severe hematopoietic toxicity in 5 out of 11 patients with T-cell lymphoproliferative disorder [61]; human IgG has a long half-life, which is around 3 weeks in serum [62]; and drug resistance, such as resistance to Rituximab, develops due to alterations in CD20, changes in membrane lipid raft domain, altered signaling pathways, and dysregulation in the mitochondrial pathway [63]. Furthermore, mAbs have a high treatment cost. 

### 5.2. Inhibiting or Depleting B Cells

Bregs play a pivotal role in the modulation of immune responses. Treating patients with CLL with an anti-CD20 mAb (Rituximab) leads to the accumulation of Bregs and lymphoma-resistant cells. IL-10-producing cells were found to positively correlate with esophagus cancer [64]. The direct depletion of Bregs by using anti-IL10 antibody treatment or the indirect depletion of Bregs by chemicals such as resveratrol has shown promise with in vitro breast tumor cell lines (4T1). Lipoxin A4 (lipid mediator lipoxin A4) and MK866 have an indirect effect on the conversion of naïve B cells into Bregs and help in decreasing B16-F10 tumor growth [42]. 

### 5.3. Activated B Cells to Suppress Tumor Growth

The CD40-CD40L costimulatory interaction activates B cells and causes the activation of cytotoxic T cells, which suppresses tumor growth. Moreover, this costimulation activates both naïve and memory T-cell components. Similarly, the stimulation of TLR-9 via CpG-ODN stimulation also causes the activation of B cells and has been shown to be effective in B6-F10 melanoma. The combined use of GM-CSF and IL-4 known as Fusiokine GIFT4 promotes B-cell activation and proliferation, and it is effective in melanoma-related tumor growth [42]. 

### 5.4. Tertiary Lymphoid Structure (TLS)

TLS is composed of CD20+ B cells and CD8+ T cells, which infiltrate tumors, and its presence correlates with patient survival during immunotherapy. Reports have validated this in metastatic melanoma [65] and sarcoma [66]. TLS has been identified as a prognostic area in breast cancer and colorectal cancer [67]. 

### 5.5. Immunotherapy Based on Tumor-Associated Autoantibodies

Autoantibody profiles in tumors can serve as tumor prognostic markers as reported in the sera of patients with breast cancer [68]. Tumor-associated autoantigens derive from either post-translational modification or aberrant overexpression. Another report suggests that P^53^ (a tumor suppressor protein) autoantibodies are associated with increased survival in hepatocellular carcinoma, while in other cancers, such as lung, colon, breast, and oral cancer, P^53^ is associated with poor survival [69]. These autoantibodies can also be used for therapeutic purposes. These autoantibodies have a variety of functions, such as ADCC, CDC, the cross-presentation of tumor antigens, and T-cell activation. Hansen et al. demonstrated that the 3E10 autoantibody characterized in lupus disease has the potential to sensitize tumor cells in in vitro cultures and human tumor xenografts to doxorubicin and/or radiation [70]. 

### 5.6. B-Cell-Epitope-Based Vaccine

This is a new avenue of B-cell-based immunotherapy, which is cost effective, safe, and makes use of polyclonal antibody responses. It involves the use of the chimeric B-cell epitope peptide receptor along with the T-cell epitope. Examples of B-cell peptide epitopes include various tyrosine kinases, such as VEGF, HER-1, HER-3, and IGF-1R [44]. An example of a B-cell-epitope-based vaccine is the HER-2/nu peptide-based vaccine used in breast and ovarian cancer. In this case, HER-2/nu is a tumor antigen [71]. Examples of the combined usage of peptide antibodies in cancer therapeutics are listed in Table 2.

Another alternative strategy is the combined usage of CD40-activated antigen-specific B cells, which can serve as APCs, along with antigen-specific plasma cells, which showed anti-tumor responses and tumor reduction in a mice model [72]. 

### 5.7. Role of Immunoglobulin in Tumor Therapy

Immunoglobulins are secreted by both cancer cells and B cells. Cancer-derived immunoglobulins show limited diversity. They promote tumor growth by inducing inflammation and the activation of platelet aggregation, and by escaping the infiltration of tumor cells. However, immunoglobulins derived from B cells are highly variable, are derived from VDJ recombination during B-cell development, and have tumor suppressor activity. The role of IgG has been defined in tumor differentiation and metastasis. IgG also has prognostic value in lung, colon, pancreatic, liver, gastric, ovarian, bladder, renal, salivary gland, soft tissue, thyroid, and parathyroid cancers. Both IgG and IgA have been shown to play important roles in oral cancer, nasopharyngeal cancer, cervical cancer, and breast cancer, as IgA primarily secretes at mucosal sites [73]. Cancer-derived cells secrete immunoglobulins that can be in either heavy chains or light chains, have both O- and N-linked aberrant glycosylation sites, have different regulatory mechanisms, and are less active [74]. 

### 5.8. Role of Cytokines and Their Association with Tumorigenesis

Bregs secrete immunosuppressive cytokines, such as IL-10 and TGF-β. IL-10 suppresses the function of cytotoxic cells, such as CD8+ T cells, NK cells, and Th1 cells, while TGFβ promotes the differentiation of B cells into IgA plasma cells, which also secrete IL-10 and express immunomodulatory receptors, such as PD-L1 FAS-L, and further suppress cytolytic activity. Oxaliplatin is a chemotherapeutic drug that can only mediate its anti-tumor activity in mouse prostate cancer when B cells are depleted, because Bregs induce TGF-β expression [24]. 

## 6. Conclusions

The role of B cells in immunotherapy is still controversial, and its potential in the field of immunotherapy has not been fully realized. This strategy of utilizing B cells in immunotherapy requires more extensive research and improvisation. B cells can have multifaceted roles in the tumor microenvironment, and depending on the cancer type and external milieu, B cells help in clearing tumors by both direct and indirect mechanisms. The direct pathway is where B cells differentiate into plasma cells and produce antibodies. Tumor-specific antibodies against neoantigens are a powerful way to kill tumor cells in a specific manner, and they have considerably fewer side effects. In the indirect method, B cells help T cells to carry out anti-tumorigenic activity. Infiltrating B cells in a tumor can help CD4+ helper T cells and CD8+ cytotoxic T cells undergo activation and expansion. Adoptive B transfer for tumor immunotherapy is still in its infancy. Ou et al. reported that B cells played significant roles in metastasis in bladder cancer by participating in IL-8/androgen receptor signaling [75]. Future work requires the identification of various B-cell subsets in cancer types and how to specifically target one subset of B cells, as complete depletion using anti-CD20 or Breg depletion is not a successful strategy to treat cancer cells completely; it leads to more side effects and toxicity. There is a need to identify biomarkers to carry out B-cell subset categorization. Another issue with using B-cell-based therapy is autoantigens. Autoantigens are expressed on both cancer cells and unmutated host cells. Therefore, targeting autoantigens can lead to the development of toxic side effects. The role of infiltrating B cells in tumors also needs to be extensively defined, as these cells actively migrate and release cytokines, and they show anti-tumorigenic effects or can have a bystander effect by producing cytokines [24]. In a nutshell, B-cell-based immunotherapy provides an alternative and promising strategy to specifically target tumor cells, but we need to carry out mechanistic studies to explore the full potential of B cells in tumor immunotherapy.

## Figures and Tables

**Table 1:** Detailed description of clinical trials completed on the role of immunoglobulin in cancer.

S. No.	Drug	Cancer	Intervention	NCT Number	Phase Trial
1.	Tumor-derived immunoglobulin idiotype antigen vaccines	B-cell lymphoma Follicular lymphoma Lymphoma	Id-KLH vaccine GM-CSF	NCT00001512	Phase 1 (National Cancer Institute)
2.	Idelalisib in combination with chemotherapeutic agents, immunomodulatory agents, and anti-CD20 mAb	Indolent non-Hodgkin’s lymphoma, chronic lymphocytic leukemia, and mantle cell lymphoma	Idelalisib, Rituximab, Bendamustine, Ofatumumab, Fludarabine, Everolimus, Bortezomib, Chlorambucil, and Lenalidomide	NCT01088048	Phase 1 (Gilead Sciences)
3.	Atezolizumab + immunomodulatory agents	Acute myeloid leukemia	Atezolizumab and Guadecitabine	NCT02892318	Phase 1 (Hoffmann-La Roche)
4.	TF2 + radio immunotherapy	Small-cell lung cancer CEA-expressing non-small-cell lung carcinoma (NSCLC)	Antibody TF2 radiation: IMP-288-Lutetium Radiation: IMP-288-Indium	NCT01221675	Phase 1 Phase 2 (Centre René Gauducheau)
5.	Oregovomab (antibody) + chemotherapy	Ovarian neoplasms	Carboplatin and Paclitaxel Biological: Oregovomab	NCT01616303	Phase 2 (Quest PharmaTech Inc.)
6.	CD40 agonistic mAbs APX005M	NSCLC, melanoma, urothelial carcinoma, MSI-H, and head and neck cancer	APX005M	NCT02482168	Phase 1 (Apexigen, Inc.)
7.	BMS-986156 +/− Nivolumab	Solid tumors	BMS-986156 and Nivolumab	NCT02598960	Phase 1 Phase 2 (Bristol-Myers Squibb)
8.	Intramuscular administration of autologous total IgG	Human cancers	Advanced solid tumor	NCT03695757	Phase 1 Phase 2 (Ajou University School of Medicine)
9.	Ipilimumab	High-risk stage III melanoma	Ipilimumab and placebo	NCT00636168	Phase 3 (Bristol-Myers Squibb)
10.	Carbo/Caelyx or Carbo/Doxorubicin with Tocilizumab (mAb IL-6R) and Peg-Intron	Recurrent ovarian cancer	Tocilizumab and interferon alpha 2-b, and Carboplatin with Caelyx or Doxorubicin	NCT01637532	Phase 1 Phase 2 (Leiden University Medical Center)
11.	Immunostimulant antibody in combination with chemotherapy	Pancreatic neoplasm	mAb chemotherapy	NCT00711191	Phase 1 (Hoffmann-La Roche)
12.	Edrecolomab	Mucinous adenocarcinoma of the colon, signet ring adenocarcinoma of the colon, stage IIA colon cancer, stage IIB colon cancer, and stage IIC colon cancer	Edrecolomab laboratory biomarker analysis	NCT00002968	Phase 3 (National Cancer Institute)
13.	Rituximab	Lymphoma	Autologous immunoglobulin idiotype-KLH conjugate vaccine Sargramostim	NCT00071955	Phase 2 (Genitope Corporation)
14.	Combination of Bevacizumab and Allogeneic NK immunotherapy	Malignant solid tumor	Bevacizumab NK immunotherapy	NCT02857920	Phase 1 Phase 2 (Fuda Cancer Hospital, Guangzhou)
15.	Belantamab mafodotin	Multiple myeloma	Belantamab mafodotin	NCT04177823	Phase 1 (GlaxoSmithKline)
16.	MOv18 IgE, chimeric IgE	Human cancers	MOv18 IgE	NCT02546921	Phase 1 (Cancer Research UK)
17.	CD40 agonistic antibody APX005M + Nivolumab	Metastatic non-small-cell lung cancer, metastatic melanoma, and neoplasm of lung melanoma	APX005M Nivolumab	NCT03123783	Phase 1 Phase 2 (Apexigen, Inc.)
18.	Galunisertib (LY2157299) and Durvalumab (MEDI4736)	Metastatic pancreatic cancer	Galunisertib Durvalumab	NCT02734160	Phase 1 (Eli Lilly and Company)
19.	Chemoembolization or ablation	Hepatocellular cancer, biliary tract neoplasms, liver cancer, hepatocellular carcinoma, and biliary cancer	Tremelimumab RFA TACE Cryoablation	NCT01853618	Phase 1 Phase 2 (National Cancer Institute)
20.	CT-011 in combination with Rituximab	Lymphoma	CT-011 Rituximab	NCT00904722	Phase 2 (M.D. Anderson Cancer Center)
21.	²¹²Pb-TCMC-Trastuzumab radio immunotherapy	Breast neoplasms, peritoneal neoplasms, ovarian neoplasms, pancreatic neoplasms, and stomach neoplasms	²¹²Pb-TCMC-Trastuzumab Biological: Trastuzumab	NCT01384253	Phase 1 (Orano Med LLC)
22.	Vaccine and antibody treatment	Prostatic neoplasms	PROSTVAC-V/TRICOM PROSTVAC-F/TRICOM MDX-010 Sargramostim	NCT00113984	Phase 1 (National Cancer Institute)
23.	FATE-NK100 as monotherapy and in combination with mAbs	HER2-positive gastric cancer, colorectal cancer, head and neck squamous cell carcinoma, EGFR-positive solid tumor, advanced solid tumors, HER2-positive breast cancer, hepatocellular carcinoma, non-small-cell lung cancer, renal cell carcinoma, pancreatic cancer, and melanoma	FATE-NK100 Cetuximab Trastuzumab	NCT03319459	Phase 1 (Fate Therapeutics)
24.	Radiation and mAbs to OX40 (MEDI6469)	Metastatic breast cancer Lung metastases Liver metastases	MEDI6469	NCT01862900	Phase 1 (Providence Health & Services)
25.	Toripalimab	Malignant lymphoma	Toripalimab	NCT03316144	Phase 1 (Shanghai Junshi Bioscience Co., Ltd.)
26.	Valproate prior to immunotherapy targeting CD20	Chronic lymphocytic leukemia	Valproate	NCT02144623	Early Phase 1 (Lund University Hospital)
27.	Ublituximab in combination with Lenalidomide	Non-Hodgkin’s lymphoma, chronic lymphocytic leukemia, small lymphocytic lymphoma, B-cell lymphomas, marginal zone lymphoma, mantle cell lymphoma, and Waldenstrom’s macroglobulinemia	Ublituximab Lenalidomide	NCT01744912	Phase 1 Phase 2 (TG Therapeutics, Inc.)
28.	JTX-2011 alone and in combination with anti-PD-1 or anti-CTLA-4	Human cancers	JTX-2011 Nivolumab and Ipilimumab Pembrolizumab	NCT02904226	Phase 1 Phase 2 (Jounce Therapeutics, Inc.)
29.	Motolimod, Doxorubicin, and Durvalumab	Ovarian cancer	Durvalumab Pegylated Liposomal Doxorubicin Motolimod	NCT02431559	Phase 1 Phase 2 (Ludwig Institute for Cancer Research)

Abbreviations: NCT: National Clinical Trial number. Clinical trial details were collected from https://www.clinicaltrials.gov/ (accessed on 22 May 2022).

**Table 2:** Combined usage of peptide antibodies in cancer therapeutics.

Combination Peptide Antibodies	Cancer Treatment
αHER-2 + αIGF-1R	Breast cancer
HER-2 + HER-3	Breast, pancreatic, and colon cancer
HER-3 + EGFR	Breast cancer
HER1 + HER2	Colorectal cancer
HER1-418 + IGF-1R-56	Pancreatic cancer
HER1-418 + HER-3-461	Pancreatic cancer

## Data Availability

There are no supporting data associated with this article.

## Abbreviations

tumor-infiltrating B cell (TIB); circulating immune complexes (CICs); acute myeloid leukemia (AML); hepatocellular carcinoma (HCC); spleen tyrosine kinase (SYK); proliferator-activated receptor α (PPARα)
